# Multifunctional Paper-Based Analytical Device for *In Situ* Cultivation and Screening of *Escherichia coli* Infections

**DOI:** 10.1038/s41598-018-38159-1

**Published:** 2019-02-07

**Authors:** Julaluk Noiphung, Wanida Laiwattanapaisal

**Affiliations:** 1 Graduate Program in Clinical Biochemistry and Molecular Medicine, Faculty of Allied Health Sciences, Chulalongkorn University, Patumwan, Bangkok 10330 Thailand; 2 Department of Clinical Chemistry, Faculty of Allied Health Sciences, Chulalongkorn University, Patumwan, Bangkok 10330 Thailand; 3 Electrochemistry and Optical Spectroscopy Center of Excellence (EOSCE), Chulalongkorn University, Bangkok, 10330 Thailand

## Abstract

Point-of-care testing (POCT) for uropathogen detection and chemical screening has great benefits for the diagnosis of urinary tract infections (UTIs). The goal of this study was to develop a portable and inexpensive paper-based analytical device (PAD) for cultivating bacteria *in situ* and rapidly testing for nitrite on the same device. The PAD was fabricated using a wax printing technique to create a pattern on Whatman No. 1 filter paper, which was then combined with a cotton sheet to support bacterial growth. Nitrite detection was based on the principle of the Griess reaction, and a linear detection range of 0–1.6 mg/dL (R^2^ = 0.989) was obtained. Scanning electron microscopy (SEM) analysis demonstrated that the bacteria were able to grow and formed a cluster on the cellulose fibres within 2 hours. The enzyme β-glucuronidase, which is specifically produced by *Escherichia coli*, was able to convert the pre-immobilized 5**-**bromo-4-chloro-3-indolyl-β-D-glucuronide sodium salt (X-GlcA), a colourless substrate, generating a blue colour. Under optimum conditions, the proposed device allowed bacterial concentrations in the range of 10^4^–10^7^ colony forming units (CFU)/mL to be quantified within 6 hours. Moreover, the use of this device enables the identification of *E*. *coli* pathogens with selectivity in real urine samples. In conclusion, the PAD developed in this study for UTI screening provides a rapid, cost-effective diagnostic method for use in remote areas.

## Introduction

In the developing world, infectious diseases are the most common cause of illness, resulting in more than 1.2 million deaths each year in those countries^1,2^. The development of simple, inexpensive, robust and portable point-of-care diagnostic devices for the early detection of infectious diseases remains an urgent need for use in most developing countries^1–4^. To cover the guidelines recommended by the World Health Organization (WHO)^5^, the ideal diagnostic test should follow the ASSURED criteria, including being affordable, sensitive, specific, user-friendly, rapid and robust, equipment-free and deliverable^5^. Among all the types of point of care diagnostic devices, paper-based sensors have become attractive and promising to meet the ASSURED criteria, because as paper is cost effective, flexible and biocompatible^1,5–7^. Moreover, microfluidic paper-based analytical devices (µPADs) have received particularly interest for detecting various types of analytes, including biological fluid biomarkers^8–10^, pathogens and contamination^11–16^, and metal compound monitoring^17^. For pathogen monitoring, contamination caused by foodborne and/or pathogens are also a significant public health issue^18,19^, and bacterial infections, such as blood, urinary tract, and respiratory tract infections are regarded as a major cause of morbidity and mortality^20^. Thus, the development of a rapid test for the early detection of bacterial infections would be valuable for the diagnosis of such infectious diseases.

Urinary tract infections (UTIs) are one of the most frequent hospital-acquired infections and are caused by a wide range of pathogens, including bacteria, fungi, viruses and parasites^21^. For the global burden of disease study 2016, the interstitial nephritis and urinary tract infections affected health loss more than 4 million people in 195 countries and territories^22^. Members of the family Enterobacteriaceae are gram-negative bacilli that are the most common cause of UTIs, with *E*. *coli* being the most common pathogen causing both uncomplicated and complicated UTIs^21^. To date, the gold standard for the diagnosis of UTIs requires both a physical examination and a microbiological assay in urine culture^23–25^. The presence of bacterial cells above 10^5^ CFU/mL, together with the detection of inflammatory cells in sterile urine, is clinically significant for UTIs^24^. In addition to a microbiological test, nitrite and leukocyte esterase testing have been used to confirm *E*. *coli* infection^25,26^. Although the conventional methods used to diagnose UTIs are widely used in most clinical laboratories, the development of an alternative method that is faster and easier would be a significant advancement^27^. The long incubation time, at least 1–2 days, is a major shortcoming of the conventional culture method. This long incubation time contributes to the delay of treatment and the spread of infectious disease, leading to the misuse of antibiotics and the development of antibiotic resistance^28^. Novel approaches enabling faster bacterial analysis must be able to accurately identify pathogens, which would contribute to the effectively antimicrobial therapy^25,28^.

Currently, rapid bacterial detection methods, e.g., the FLEXICULT™ SSI-Urinary Kit, are available in the market^29^. However, this kit is rather expensive and still requires one day for bacterial culturing. To date, molecular biology techniques have been used to detect microorganisms and in epidemiological studies^30–32^, such as multiplex PCR methods used to detect *E*. *coli* serogroups^31^. Nevertheless, molecular techniques are limited to the laboratory and should be performed in a closed system to prevent contamination^30,32^.

The use of paper^7^ and/or other types of biocompatible substrates, such as cotton threads^33,34^, cloth^35^, cotton^36^ and lignocellulose^37^ have become attractive in biosensors research because of their flexibility and cost effectiveness. Several studies have reported on the use of paper-based analytical devices for the quantitative analysis of nitrite and nitrate based on colourimetric assays^8,38–40^. These assays can be used to quantify the target analytes in variety type of samples, such as saliva^8,38^ and drinking water^39^. However, previous reports have not focused on the development of sensors for monitoring UTIs^8,38–40^. A paper-based device has been reported for the culturing and identification of bacteria based on the T4 bacteriophage infection of *E*. *coli* cells and the detection of released β-galactosidase, and this device has been used for environment monitoring^41^. A major drawback of this method is the utilization of T4 bacteriophage, which is known to infect only 60% of *E*. *coli* strains^41^, raising the possibility of false negative results being obtained. To pre-concentrate the bacteria from complex sample matrices, immunomagnetic separation (IMS) has been utilized in which samples are mixed with antibody-attached beads to capture the cells of interest^11,42^. Combining IMS with paper-based devices has been reported for the detection of *E*. *coli*^42^ and *S*. *typhimurium*^11^ in complicated sample matrices. To detect UTIs and gonorrhoea on µPADs, the principle used is based on the immunoagglutination of antibody-conjugated particles and the specific targeting and detection of nitrite using a commercial strip. The detection limit determined for both *E*. *coli* and gonorrhoea using this method was 10 CFU/mL^43^. In addition to their use as PADs, lateral flow test strips have been successfully developed for multiplex analysis of whole bacterial cells, which were applied in point-of-care diagnostics tests because these tests allow real-time monitoring, simultaneous detection and short analysis times^44,45^. A significant benefit of immunoassays is the high specificity and high sensitivity for detecting low concentrations of bacteria. However, the limitation of antibody-based bacterial detection is its inability to distinguish between living and dead cells^41^. The differentiation of live or dead cells is regarded as an important requirement for antibiotic treatment. Recently, the use of PADs to detect β-lactamase producing bacteria based on the reaction between β-lactamase and nitrocefin was demonstrated and was used to detect β-lactam resistance in wastewater and sewage^12^. The properties of PADs make them useful as simple and low-cost platforms for bacterial growth^46^, identification^14,16^ and susceptibility testing^15^. However, only a few studies have described the development of PADs for both the cultivation and identification of bacterial species and for their use in screening for urinary tract infections.

The goal of this study was to develop a new, simpler PAD for the simultaneous detection of nitrite and bacterial cultivation and identification from urine samples on the same device. This is the first report on the use of Whatman No. 1 filter paper with cotton pads for supporting bacterial growth on a PAD. The moisture absorption and biocompatibility^36^ qualities of cotton make it an excellent choice of materials for supporting bacterial growth. To allow for rapid screening of gram-negative bacteria, nitrite detection based on the Griess reaction^47^ was utilized. In concurrence with nitrite detection, a biochemical test for the production of the β-glucuronidase was also included in the PAD. Because approximately 95% of *E*. *coli* strains release β-glucuronidase^48^, our proposed PAD should be specific for *E*. *coli* detection in clinical samples. Importantly, the colour change on the culture area can be visually detected. Because the bacterial isolation step is omitted using this device, our proposed PAD has promise for use in the rapid screening of UTIs, especially in remote areas.

## Results

### Fabrication and characterization of paper-based analytical device (PAD)

**Figure 1:**
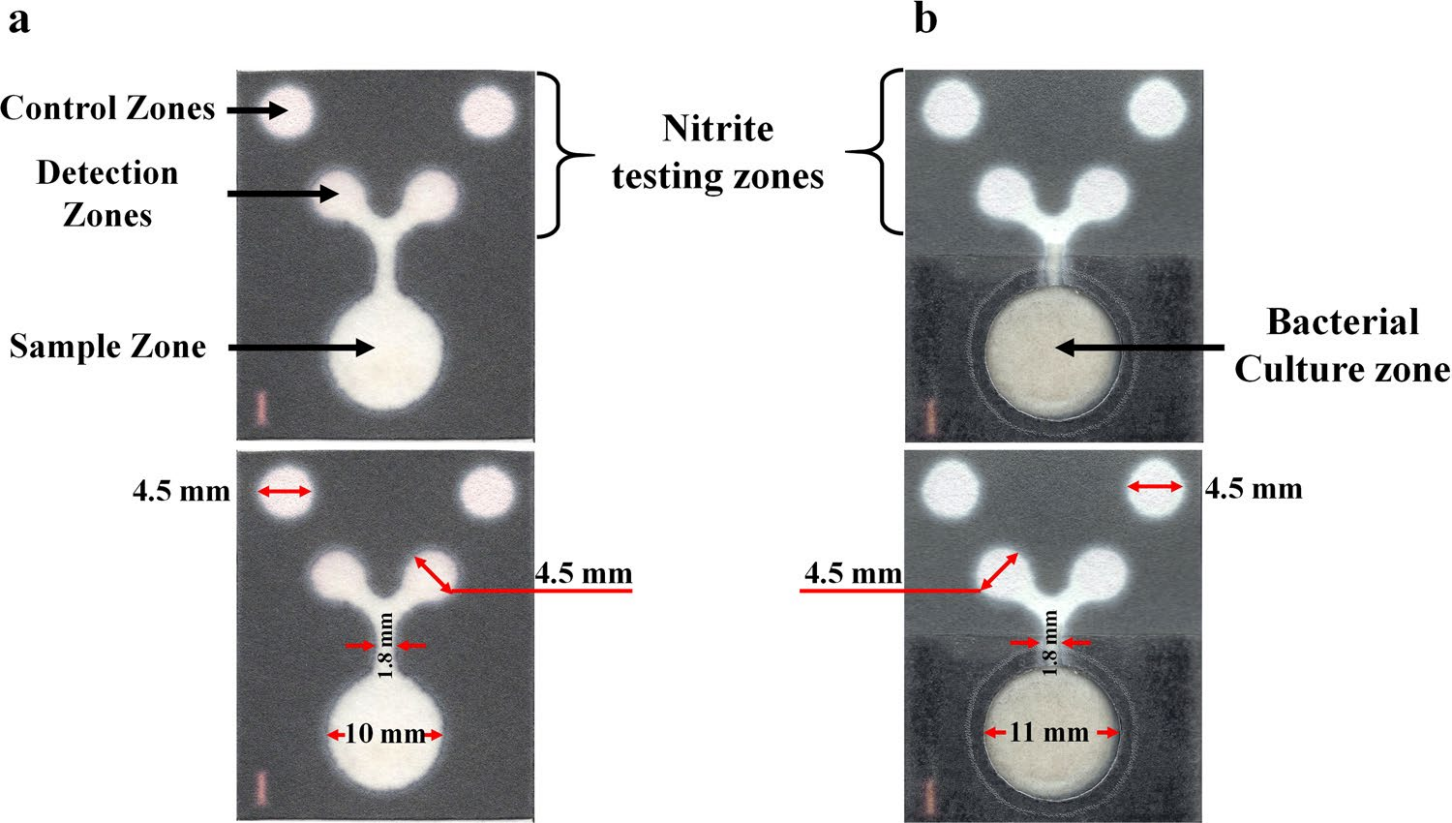
**(a**) Design of a paper-based analytical device (PAD) for nitrite quantification (Top). The dimensions of testing area in the PAD after heating and sterilization (Bottom). (**b**) Design of the multifunctional PAD for nitrite determination and bacterial cultivation and identification (Top). The dimensions of the PAD testing area after heating and sterilization (Bottom).

During the PAD fabrication process, a hydrophobic area was created using a wax printing technique, and the prototype PAD for nitrite determination is shown in Fig. 1a. To allow for the multiple functions of the PAD, the printed papers were successfully combined with the cotton pad, in which the latter was intended for use as the supporting area for bacterial cultivation (Fig. 1b). The PAD fabrication process is illustrated in Supplementary Fig. S1.

The surface properties of the paper before and after sterilization were characterized using Field Emission Scanning Electron Microscopy (FESEM). To avoid contamination, the selection of a sterilization process is very important and is inevitably performed on every PAD before used. In this study, an autoclave was used to sterilize the PADs. The high temperature reached during sterilization affected the wax melting, leading to a narrowing of the hydrophilic flow channel that resulted in a non-uniform pattern formation^49^. To solve this problem, clear packing tape was attached to one side of paper and then was placed in a heat-resistant plastic bag before being autoclaved. Interestingly, the attachment of the packing tape underneath the paper resulted in a uniformly patterned PAD as shown in Supplementary Fig. S2.

**Figure 2:**
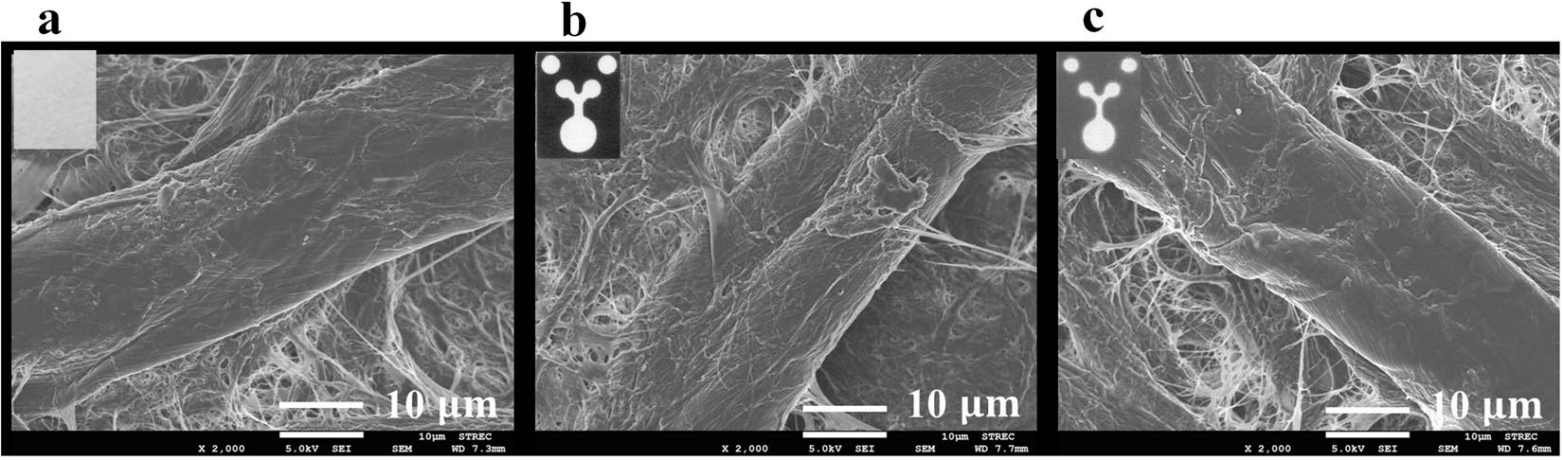
Field Emission Scanning Electron Microscopy (FESEM) analysis. (**a**) Whatman No. 1 filter paper, (**b**) Whatman No. 1 filter paper heated at 150 °C for 2 minutes, and (**c**) Whatman No. 1 filter paper heated at 150 °C and autoclaved at 121 °C for 20 minutes. These images are representative of triplicate experiments.

To confirm the surface morphology of the cellulose-based paper after exposure to high temperature, three types of surface paper treatment were performed and assayed by FESEM, including (1) a plain Whatman No. 1, (2) Whatman No. 1 heated at 150 °C for 2 minutes and (3) Whatman No. 1 heated at 150 °C for 2 minutes and autoclaved at 121 °C for 20 minutes. Figure 2 displays the microstructures of the three different papers as determined by the FESEM analysis. The visual assessment suggests that heating the paper did not affect the structure of the paper device.

### Standard curve of nitrite and real sample analysis

**Figure 3:**
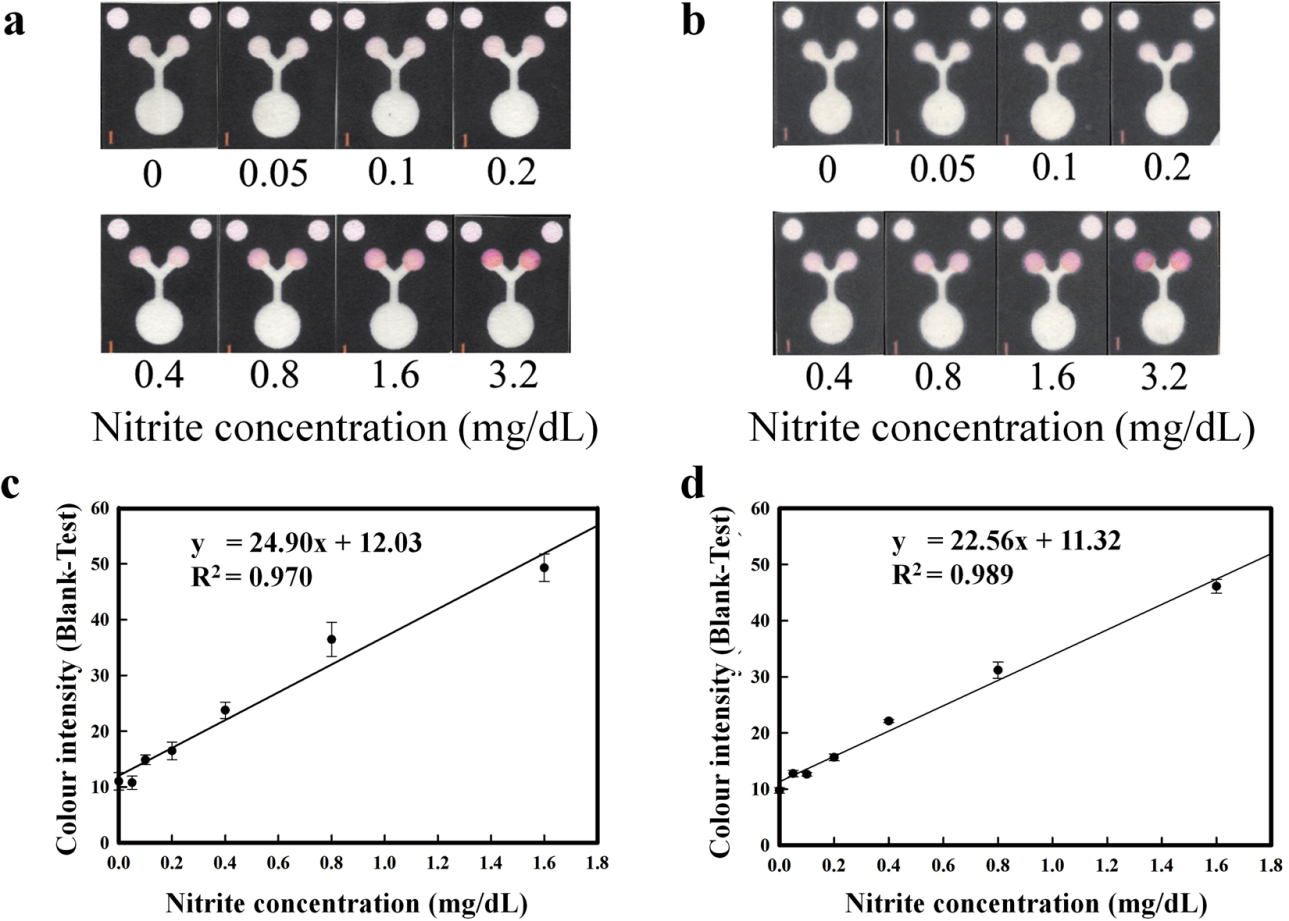
(**a**,**b**) Colour changes on the PAD corresponding to different concentrations of nitrite. (**c** and **d**) Analytical calibration curves representing the linear range of nitrite from 0 to 1.6 mg/dL (n = 4). ((**a**,**c**) show data from Whatman No. 1 filter paper, while (**b**,**d**) show data from autoclaved Whatman No. 1 filter paper).

The first pattern of papers (Fig. 1a) was used to generate a standard curve for nitrite and to quantify the nitrite level in the urine control. The nitrite test was based on the Griess reaction, wherein nitrite reacts with sulfanilamide under acidic conditions to form a diazonium salt and then couples to N-(1-naphthyl)-ethylenediamine dihydrochloride (NED) to produce a red-pink colour^47^. NED is photosensitive and changes to pink when exposed to ambient light. To overcome this limitation, control areas were created on the proposed paper, and the mean colour intensity of each concentration was calculated by subtracting the intensity of the testing areas from the intensity of the control areas^8^. Figures 3a,b display a colour change on the paper-based device that is proportional to the nitrite concentration. The curve shows increasing colour intensity with increasing nitrite concentration. The linear function of this curve is y = 24.90x + 12.03, R^2^ = 0.970 for Whatman No. 1 filter paper and y = 22.56x + 11.32, R^2^ = 0.989 for sterilized Whatman No. 1 filter paper, as shown in Fig. 3c,d. The data from the FESEM analysis and the slope of linear function of the standard curve indicate that the sterilization process did not affect the surface morphology of the cellulose paper or the nitrite measurement.

For real sample analysis, urine controls (level 1) spiked with various concentrations of nitrite were used to test the performance of the sensor, and the measured nitrite values are shown in Table 1. Surprisingly, when using volumes less than 20 µL, the nitrite concentrations in the control urine did not correlate well with spiked concentrations. These inconsistent results may have occurred because of the difference in viscosity between water and the control urine, particularly when an insufficient sample volume was used. The nitrite standard dissolved in distilled water has a specific gravity of 1, whereas the MAS^®^ UA control (level 1) has a specific gravity of approximately 1.005–1.020 (Manufacturer leaflet of MAS^®^ UA control, Thermo Scientific, MA, USA). Therefore, the time required for the standard nitrite solution and the spiked control urine to reach the detection zones was different. This difference led to the reaction time of the control urine being shorter than those of standard nitrite solutions, leading to lower concentrations being calculated. This phenomenon has also been observed with respect to tear fluid viscosity^50^ and saliva viscosity^8^. A good correlation was obtained for the control urine at sample volumes of 20 µL and higher. However, when using volumes 22 or 25 µL, the PADs became soaked and required assay times longer than 10 min until the PADs were ready for imaging. Because the sample zone became overloaded when 30 µL of sample was used, this volume was not suitable for this size of PAD (Supplementary information, Fig. S3). Therefore, a sample volume of 20 µL was used for the subsequent experiments.

**Table 1:** Analysis of nitrite in a urine control spiked with different concentrations of nitrite using the paper-based analytical device (n = 4).

Added nitrite standard	Sterilized Whatman No. 1 paper		
	13-µL sample	18-µL sample	20-µL sample
0 mg/dL	0 mg/dL	0 mg/dL	0 mg/dL
0.5 mg/dL	0.29 ± 0.07 mg/dL	0.39 ± 0.07 mg/dL	0.60 ± 0.08 mg/dL
1.0 mg/dL	0.50 ± 0.05 mg/dL	0.89 ± 0.18 mg/dL	1.10 ± 0.05 mg/dL

The reproducibility of the sensor for measuring nitrite was evaluated at nitrite concentrations of 0.5 and 1.0 mg/dL, and coefficients of variation (CVs) of 9.07% (0.58 ± 0.05) and 7.90% (1.02 ± 0.08) (n = 20) were observed, respectively. These results demonstrate that the proposed PAD has the capability of quantifying nitrite in urine samples.

### Optimum concentration of 5-bromo-4-chloro-3-indolyl-β-D-glucuronide sodium salt (X-GlcA) substrate

**Figure 4:**
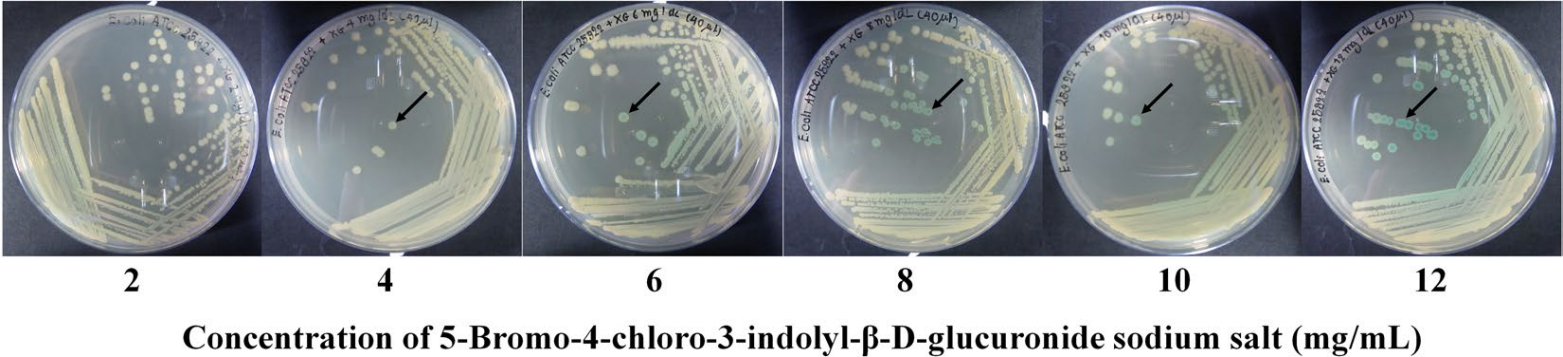
Blue pigmented colonies after bacterial growth on agar plates with various concentrations of the X-GlcA substrate.

The hydrolytic properties of the β-glucuronidase enzyme from *E*. *coli* with the X-GlcA substrate were studied on LB agar. The colour change of bacterial colonies on agar from colourless to blue can be observed with the naked eye at substrate concentrations ranging from 4 to 12 mg/mL. Figure 4 shows the generation of blue colonies in the presence of various concentrations of the X-GlcA substrate. The substrate concentrations that produced deep blue colonies, i.e., 6, 8, 10 and 12 mg/mL, were selected for further testing with the proposed PAD.

Subsequently, the optimum X-GlcA substrate concentration and reaction time on the proposed paper were studied using the proposed device. For the sensor, colour changes could be observed at 4 hours for all substrate concentrations. An X-GlcA concentration of 10 mg/mL and a reaction time of 6 hours were selected for the generation of analytical curves for bacterial detection. The colour intensities of the paper devices using varying substrate concentrations and incubation times are shown in Supplementary Fig. S4.

### Field Emission Scanning Electron Microscopy (FESEM) analysis of bacterial culture on the PAD

**Figure 5:**
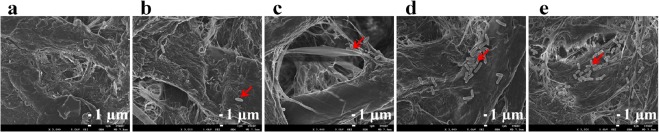
Field emission scanning electron microscopy (FESEM) analysis of bacterial growth on Whatman No. 1 filter paper. (**a**) Luria-Bertani (LB) broth without bacteria. (**b**) A total of 10^4^–10^5^ CFU/mL of *E*. *coli* after 0 hours of culturing on the PAD. (**c–e**) A total of 10^4^–10^5^ CFU/mL of *E*. *coli* after 1, 2 and 3 hours of culturing on the PAD. The red arrow in (**b–e**) indicates bacterial cells on the cellulose fibre. These images are representative of triplicate experiments.

FESEM analysis was used to ensure the growth of the bacterial cells on the PAD. As shown in Fig. 5d, a visual assessment of the PAD demonstrated that the bacteria grew and formed a cluster on the cellulose fibres of Whatman No. 1 filter paper within 2 hours. The fibrous and porous properties of filter paper promote bacterial cell attachment, as shown in Fig. 5d–e. Moreover, the bacterial growth on the proposed devices was supported by the addition of a cotton pad, which has absorptive properties and provides a stable platform^36^. Therefore, it can be concluded that cellulose fibres combined with cotton and agar can be used for bacterial cultivation.

### Analytical range for *E*. *coli* detection on the proposed PAD

**Figure 6:**
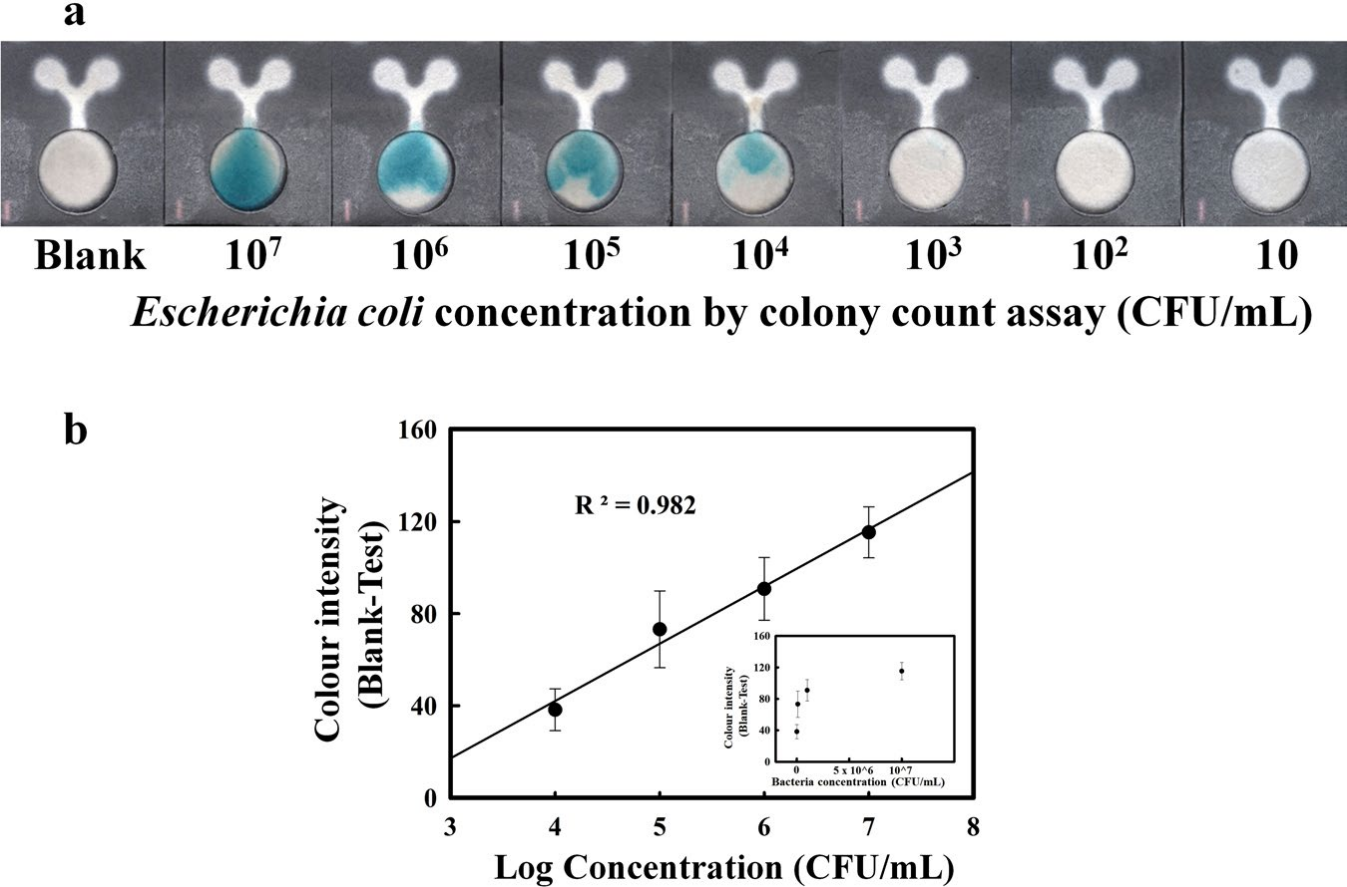
(**a**) Colour change in the presence of various concentrations of *E*. *coli* after incubated on devices for 6 hours. (**b**) Analytical curve of the relationship between colour intensity and the logarithm of the bacterial concentration (n = 4). Insets show the relationship between the colour intensity and the bacterial concentration.

Based on the intensity of the blue pigment, colour changes on the proposed device can be observed within 4 hours, which corresponds to the exponential phase of bacterial growth. The proposed device could quantify *E*. *coli* with a detection range of 10^4^–10^7^ CFU/mL within 6 hours. Moreover, below 10^4^ CFU/mL, the concentration of *E*. *coli* could be monitored by the naked eye when incubated on the device for 10 hours, as shown in Supplementary Fig. S5. The observed colour changes for different quantities of *E*. *coli* cells on the PADs are presented in Fig. 6a. Figure 6b shows the correlation between colour intensity and the logarithm of the concentration of *E*. *coli* cells, R^2^ = 0.982.

As shown in Fig. 6a, the minimum concentration of *E*. *coli* at which a change in the X-GlcA substrate from colourless to a blue colour could be visually observed by the naked eye at 6 hours was 10^4^ CFU/mL. To confirm the limit of detection (LOD) of this proposed method, a single colony of *E*.*coli* was inoculated in LB broth followed by incubation for 4 hours at 37 °C with continuous shaking. Subsequently, the 10-fold serial dilutions of bacterial solution were prepared and each bacterial suspension was portioned to culture on the proposed PADs. The actual concentration of *E*. *coli* in each suspension was determined by a standard plate counting method. The minimum concentration of *E*. *coli* able to convert the colourless X-GlcA substrate to the blue pigment as visually observed on the PAD at 6 hours was considered as the LOD of this method.

The result demonstrated that the concentration of *E*. *coli* in LB broth was 1.57 ± 0.46 × 10^7^ CFU/mL, as determined in triplicate experiments by plate counting method. After 10-fold serial dilutions, the lowest concentration of *E*. *coli* able to produce blue pigment as visually observed by the naked eye at 6 hours was 1.57 × 10^4^ CFU/mL (n = 4), as shown in the Supplementary data Fig. S6a. Interestingly, the concentrations of *E*. *coli* up to 10^3^ CFU/mL could be visually detected after 7 hours incubation, and the image is shown in Supplementary Fig. S6b.

However, based on 6 hours incubation, the detection limit of 10^4^ CFU/mL is quite high compared to that observed in other studies^41,43^. Nevertheless, the range for *E*. *coli* detection by our proposed paper sensor for screening bacteria in urinary tract infection (UTIs) covers that needed to diagnose UTIs, which requires the presence of bacterial cells above 10^5^ CFU/mL^24^. However, the proposed device could be used to monitor *E*. *coli* in single step without adding the inducer or indicator dye and omitting the bacterial isolation step. Moreover, the PAD could be combined with additional chemical screening functionalities, including for nitrite, protein and leukocyte esterase testing to confirm an infection^25,26^.

### Real sample analysis and specificity study

**Figure 7:**
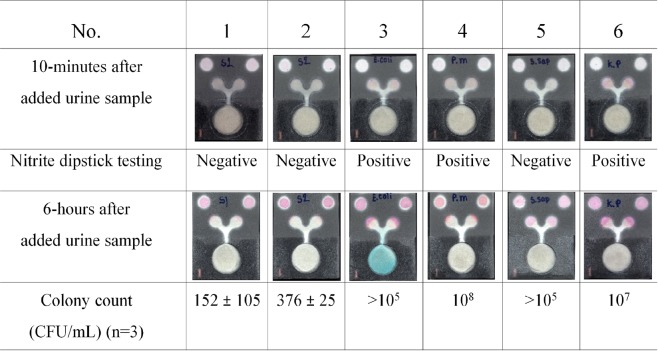
Real sample analysis using the proposed PAD for nitrite determination and bacterial cultivation and identification.

After a prototype device for nitrite determination and bacterial cultivation was successfully developed, urine samples were tested on the proposed device. As shown in Fig. 7, the nitrite detection results at the nitrite detection zones show a good correlation with the nitrite strip test assay. However, the colour change in the nitrite detection area of the PAD are paler than that observed using a strip test and the nitrite standard on PADs, which may result from the effect of sample loss in µPADs^51^. Moreover, the immersion of the paper surface in the culture area with medium resulted in a decrease in the absorption properties of the cellulose fibre. For the bacterial analysis, two urine samples from healthy subjects (samples 1 and 2) were assayed that contained approximately <500 bacterial cells as determined by a standard plate count. Samples 3, 4, 5 and 6 contained *E*. *coli*, *P*. *mirabilis*, *S*. *saprophyticus* and *K*. *pneumoniae*, respectively, and all of the urine samples contained >10^5^ CFU/mL of the assayed bacteria. As shown in Fig. 7, the blue colour in the culture area of sample 3 could be observed via the naked eye within 6 hours, whereas the samples containing other bacteria (*P*. *mirabilis*, *S*. *saprophyticus* or *K*. *pneumoniae*) could not produce the blue pigment in the proposed PAD. Therefore, it can be concluded that the proposed PAD holds considerable promise for the simultaneous determination of nitrite and *E*. *coli* in urine samples with high specificity.

## Discussion

In the present study, we describe a simple and low-cost PAD for the simultaneous determination of nitrite and for bacterial cultivation and identification in a single step. The various viscosities of biological samples have a significant impact on the fluid flow in paper channels^8^. Although, the described PAD is promising for nitrite detection, the inconsistent nitrite measurement results may result from the viscosity of the urine solution. The nitrite standard dissolved in distilled water has a specific gravity of 1, whereas the MAS^®^ UA control (level 1) has a specific gravity of approximately 1.005–1.020 (Leaflet of MAS^®^ UA control, Thermo Scientific). This phenomenon is similar to that observed for tear fluid viscosity^50^ and saliva viscosity^8^, which is particularly important when making PAD-based measurements. Previous results have revealed that the wicking distance in the paper channel depends on the time of sample flow and on the fluid viscosity^50^. This behaviour can be described by a modified Navier- Stokes equation, as shown in (1)^50^. According to this equation, the fluid viscosity can be

1 $$t=\eta {L}^{{\bf{2}}}/k{\rm{\Delta }}P$$

where *t* is the time, *η* is the viscosity, *L* is the wicking distance, *k* is the permeability of the fluid, and Δ*P* is the applied pressure difference^50^. It is generally known that Griess reagent is photosensitive and unstable when exposed to ambient light. Therefore, several studies have reported a strategy to prevent the degradation of Griess reagent, i.e., separating the components of the Griess reagent^38^ and by adding Nafion to the nitrite cocktail to avoid the leaching of the cationic azo-dye formed by the reaction^40^. However, in this study, the problem of high background colour was overcome by providing a control area on the PAD for subtracting the colour background intensity. Furthermore, the combined use of a paper-based device and a cotton pad was designed especially for supporting bacterial growth. Generally, the growth of bacteria in liquid medium is divided into 4 phases, including the lag, exponential, stationary and death phases. In the optimal culture media, *E*. *coli* adapts and divides at a constant rate for approximately 100 minutes^52^. As shown in the FESEM image, the observed rate of *E*. *coli* growth on the PAD was similar to the growth of the bacterium in liquid medium. Because both paper and cotton are biocompatible materials, they have been used as substrates for a variety of biomedical applications^53^. Although the bacteria were able to grow and formed a cluster on the PADs within 2 hours, the number of bacteria was not sufficient for monitoring by colourimetric assay. However, a detection time of 6 hours is significantly shorter than that of a conventional bacterial culture, which requires at least 24 hours for a UTI diagnosis^26^. Using our proposed devices, 10^4^–10^7^ CFU/mL of *E*. *coli* could be quantified within 6 hours. However, the limit of detection is rather high when compared to those reported in previous studies. Using a method based on β-galactosidase gene-carrying T4 bacteriophage to infect bacterial cells, less than 10 CFU/mL of *E*. *coli* can be detected within 8 hours by a colourimetric assay and within 5.5 hours by a bioluminescence assay^41^. Unfortunately, T4 bacteriophage only infects 60% of *E*. *coli* strains^41^. The purpose of this study was to develop a screening method for UTIs, and the sensitivity of the described device was sufficient to detect bacteria in range required for the diagnosis of UTIs, as bacteria were detected above 10^5^ CFU/mL^24^. The PAD design concept used in this study was similar to previous reports describing the use of a paper substrate, packing tape and PDMS membrane to fabricate culture devices^46^. However, we used low-cost material and instrumentation that is available in developing countries to create our culture device. For specificity testing, indoxyl-β-D-glucuronide chromogenic β-glucuronidase has a sensitivity of 88 to 90% and a specificity of 100% for the rapid detection of *E*. *coli* on MacConkey agar^54^. Our results showed that only *E*. *coli*, which causes more than 70% of UTIs, could change the colour of the X-GlcA substrate. The chromatic change can be observed with the naked eye, whereas fluorogenic substrates such as 4-methylumbelliferyl-β-D-glucuronide (MUG) require specific wavelengths of light for visualization^55^.

## Conclusions

In this study, a portable and cost-effective paper-based analytical device (PAD) for screening UTIs was successfully developed. The method offers an advantage of *in situ* bacterial cultivation and identification, in which the method was faster than the standard culture method^26^. The specificity of the proposed method was achieved using a β-glucuronidase-specific substrate, as approximately 95% of *E*. *coli* strains can release this enzyme^48^. Quantification of the number of bacterial cells in urine samples greatly benefits the diagnosis and prognosis of UTIs. Moreover, this platform can be used to quantify nitrite over the entire range of normal and clinically significant levels. Therefore, UTIs caused by *E*. *coli* can be confirmed using this novel PAD. The system is not limited to nitrite and bacterial detection, as it can be extended by being combined with other biochemical tests, e.g., drug susceptibility testing. Moreover, this paper-based platform can be incorporated with other detection techniques, such as fluorescence-based assays and electrochemical techniques, for rapid monitoring of bacterial cells.

## Materials and Methods

### Materials and reagents

The materials used to fabricate the PAD described in this study included Whatman No. 1 filter paper (Cat No. 1001–185), obtained from GE Healthcare UK Limited, UK, and cotton pads (Shiseido Cleansing Cotton, Shiseido, Japan), purchased from a cosmetic shop in Thailand. Adhesive tape (CROCO, clear, 72-mm) and aluminium foil were obtained from a local grocery store. The chemicals used to prepare the detection areas and to generate the calibration curve for nitrite included sulfanilamide (≥99%, CAS No. 63741), citric acid (≥99.5%, CAS No. 77929), and N-(1-naphthyl) ethylenediamine dihydrochloride (≥98%, CAS No. 1465254), as well as a nitrite standard for IC 1,000 mg/L ± 4 mg/L (Pcode No. 101693502) were purchased from Sigma-Aldrich, USA. For bacterial culture and identification, 5-bromo-4-chloro-3-indolyl-β-D-glucuronide sodium salt (≥98%, Pcode 1001943317) was purchased from Sigma-Aldrich, USA. Quality control bacterial strains, including *Escherichia coli* ATCC^®^ 25922^TM^, *Proteus mirabilis* ATCC^®^12453^TM^, *Klebsiella pneumoniae* subsp. *pneumoniae* ATCC^®^ 13883^TM^ and *Staphylococcus saprophyticus* ATCC^®^ 15305^TM^ were purchased from Microbiologics, Inc., USA. Agar A, yeast extract and tryptone powder were obtained from Bio Basic Canada, Inc., Canada. Sodium chloride (CAS No. 7647145) was purchased from Merck, Germany. Tryptic soy broth (CAS No. 63741) was obtained from Sigma-Aldrich, USA. Tryptone soy agar and nutrient agar plates were obtained from Oxoid Limited, UK. Two levels of MAS^®^ UA control (liquid assayed urinalysis control) were purchased from Thermo Scientific, USA.

### Paper-based device fabrication process

The prototype PAD consists of two regions, one for a nitrite assay and one for bacterial culturing. The PAD pattern was created by using a wax printing technique to construct hydrophobic barriers on the paper. The first prototype consisted of one sample zone (12-mm diameter) for sample application, two detection zones (6.5-mm diameter) and two control zones (6.5-mm diameter) for nitrite determination. The pattern was printed on Whatman No. 1 filter paper using a wax printer, after which the printed paper was placed on a hot plate at 150 °C for 2 minutes. Subsequently, clear packing tape was adhered to one side of the PAD prior to sterilization of the PAD by autoclaving. The dimensions of the proposed device are shown in Fig. 1a. For the nitrite assay, Griess reagent was immobilized onto the detection and control zones and was subsequently allowed to air-dry in darkness at room temperature.

In this study, the second PAD prototype consisted of an area for nitrite detection combined with an area for bacterial cultivation (Fig. 1b). An 11-mm diameter hole was cut out of the sample zone of the wax printed paper using a circle cutter, and then the back side of the entire PAD was coated with a clear packing tape. Next, a 10-mm diameter cotton pad was inserted into the hole of the PAD, where the cotton pad was placed over the packing tape. For sterilization, the PADs were placed in a heat-resistant plastic bag to protect it from penetration by water stream and then were autoclaved and allow to dry in a hot-air oven at 65 °C overnight. To prepare the bacterial culture area, 2% Luria-Bertani (LB) agar was pipetted over the cotton pad and the 5-bromo-4-chloro-3-indolyl-β-D-glucuronide sodium salt (X-GlcA) substrate was added to the Whatman No. 1 filter paper. The paper was then folded, and the device was exposed to ultraviolet (UV) light for 15 minutes before use. The PAD fabrication process is illustrated in Supplementary Fig. S1 and the pattern of the PAD is shown in Fig. 1b.

### Nitrite determination on the PAD

A stock solution of a 1,000 mg/L ± 4 mg/L (100 mg/dL) nitrite standard was diluted with milliQ water to create standard solutions at concentrations of 0.05, 0.1, 0.2, 0.4, 0.8, 1.6 and 3.2 mg/dL. Nitrite determination on the PAD was performed based on the Griess reaction. The reagent solution for the nitrite assay contains 50 mM sulfanilamide, 330 mM citric acid and 10 mM N-(1-naphthyl) ethylenediamine dihydrochloride. All the chemicals were dissolved in milliQ water and were stored in the dark. To quantify nitrite alone, 0.7 μL of the nitrite solution was immobilized in the testing and control areas and was left to dry in the dark for 15 minutes. Subsequently, 13 μL of nitrite standard was aliquoted into the sample area. After 10 minutes of colour development, the PAD was scanned using a flatbed scanner (Epson Perfection V39 colour scanner, Epson Thailand Co Ltd. Bangkok, Thailand), and the colour intensity of the image was analysed using Adobe Photoshop CC 2015. The colour intensity was calculated by measuring the magenta channel in Photoshop^®^ (CMYK mode).

### Bacterial preparation and cultivation

To optimize the concentration of the 5-bromo-4-chloro-3-indolyl-β-D-glucuronide sodium salt (X-GlcA) substrate for bacterial detection, of 2, 4, 6, 8 and 10 mg/dL solutions of the substrate were evaluated by spreading 40 μL of the solutions onto LB agar. Subsequently, *E*. *coli* ATCC® 25922TM was streaked onto the agar medium and incubated at 37 °C for 24 hours. The concentrations that produced blue pigmented colonies were then selected for testing on the proposed PAD. For bacterial detection on the PAD, the different concentrations of the X-GlcA substrate were aliquoted into the culture area of each PAD, and *E*. *coli* ATCC 25922 at 10^4^–10^5^ CFU/mL, determined based on the colony count method, was subsequently immobilized in this zone. The PAD was covered with a sterilized plastic sheet to prevent contamination and evaporation and was incubated at 37 °C. The colour change on the PAD was recorded at 2, 4, 6, and 8 hours.

### Field emission scanning electron microscopy (FESEM) analysis

To confirm the surface properties of the paper after sterilization, the surfaces of three types of paper, including Whatman No. 1 filter paper, Whatman No. 1 filter paper heated at 150 °C for 2 minutes and Whatman No. 1 filter paper heated at 150 °C for 2 minutes, affixed with packing tape and autoclaved at 121 °C for 20 minutes, were analysed via Field Emission Scanning Electron Microscopy (FESEM; JEOL, model JSM7610F, JAPAN). The papers were coated with platinum using a sputter coater (Quorum model Q150RS) and were observed by FESEM. To ensure bacterial growth on the paper, an *E*. *coli* culture on the PAD was prepared to obtain a concentration of approximately 10^4^–10^5^ CFU/mL according to the colony count method, and the solution was then applied to the culture area.

Bacteria were grown on the filter paper for 0, 1, 2 and 3 hours, fixed overnight in 2.5% glutaraldehyde in 0.1 M phosphate buffer (pH 7.2) and then were rinsed twice with phosphate buffer and once with distilled water. The samples were dehydrated by a gradient series of ethanol (30, 50, 70, and 95% for 5 minutes each and 100% three times for 5 minutes each). The dehydrated papers were dried to a critical point, coated with platinum and observed by FESEM. Filter paper with LB broth was used as a control.

### Generation of a bacterial standard curve

A standard curve of *E*. *coli* was obtained by inoculating one colony of *E*. *coli* in LB broth followed by incubation at 37 °C with shaking overnight. The bacteria were enumerated by plating 10-fold dilutions (10^−1^, 10^−2^, 10^−3^, 10^−4^, 10^−5^ and 10^−6^), and the number of bacteria was confirmed by the plate counting method. Subsequently, each bacterial culture was applied onto the PAD and incubated at 37 °C for 6 hours. The colour change on the surface of the paper was recorded using a flat scanner, and the colour intensity was measured using the cyan channel in Photoshop^®^ (CMYK mode).

### Real sample analysis and specificity test

All research work was performed in accordance with relevant guidelines of a protocol approved by the Ethics Review Committee for Research Involving Human Research Subjects, Health Science Group, Chulalongkorn University, Bangkok, Thailand. (COA No. 061.1/59). Midstream urine from healthy volunteers was collected in sterile containers. and informed consent was obtained from all subjects involved in this study. The agar plates with four types of bacteria, including *E*. *coli*, *P*. *mirabilis*, *S*. *saprophyticus* and *K*. *pneumoniae* were incubated at least 18 hours at 37 °C. A single colony of each bacterial species was picked and inoculated into sterile urine and incubated for 7–8 hours at 37 °C. Next, the nitrite standard was spiked into healthy urine to obtain a positive control as confirmed using a commercial rapid test. To perform the assay with the PAD, 20 µL of urine was added onto the culture area and then was allowed to stand for 10 minutes to observe the colour change for nitrite detection. After incubating for 6 hours at 37 °C, the PAD images were captured and analysed. The colour changes on the surface of the paper were recorded using a flat scanner.

## Supplementary information

supplementary information
