# Supplementary material for: Multifunctional Paper-Based Analytical Device for In Situ Cultivation and Screening of Escherichia coli Infections
Source: Sci Rep. 2019 Feb 7;9:1555. doi: 10.1038/s41598-018-38159-1 (PMC6367442; doi:10.1038/s41598-018-38159-1)
Supplement: Supplementary file 1 — supplementary information [file 41598_2018_38159_MOESM1_ESM.pdf]

Supplementary Data

Multifunctional Paper-Based Analytical Device for *In Situ* Cultivation and Screening of *Escherichia coli* Infections

Julaluk Noiphung[1] and Wanida Laiwattanapaisal[2,3]*

[1] Graduate Program in Clinical Biochemistry and Molecular Medicine, Faculty of Allied Health Sciences, Chulalongkorn University, Patumwan, Bangkok, 10330, Thailand
E-mail address: julaluk2304@gmail.com

[2] Department of Clinical Chemistry, Faculty of Allied Health Sciences, Chulalongkorn University, Patumwan, Bangkok, 10330, Thailand

[3] Electrochemistry and Optical Spectroscopy Center of Excellence (EOCE), Chulalongkorn University, Bangkok, 10330, Thailand

Corresponding author: Wanida.L@chula.ac.th, Phone number: +6-695-509-2205

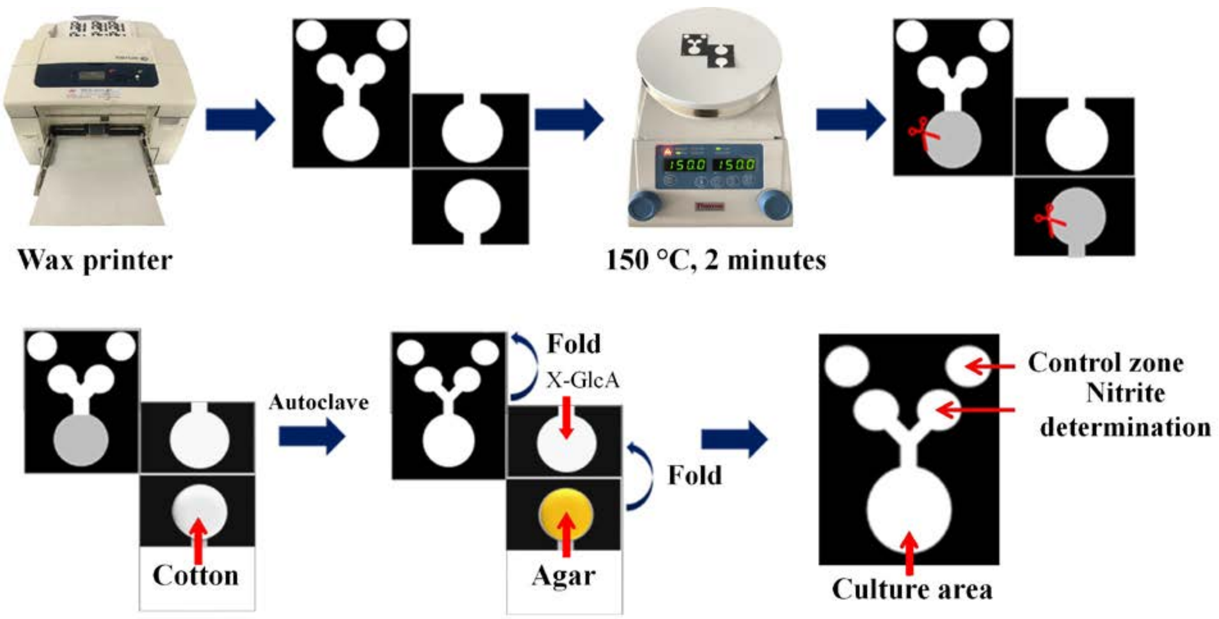

Figure S1. Fabrication process of a paper-based device for nitrite determination and bacterial cultivation.

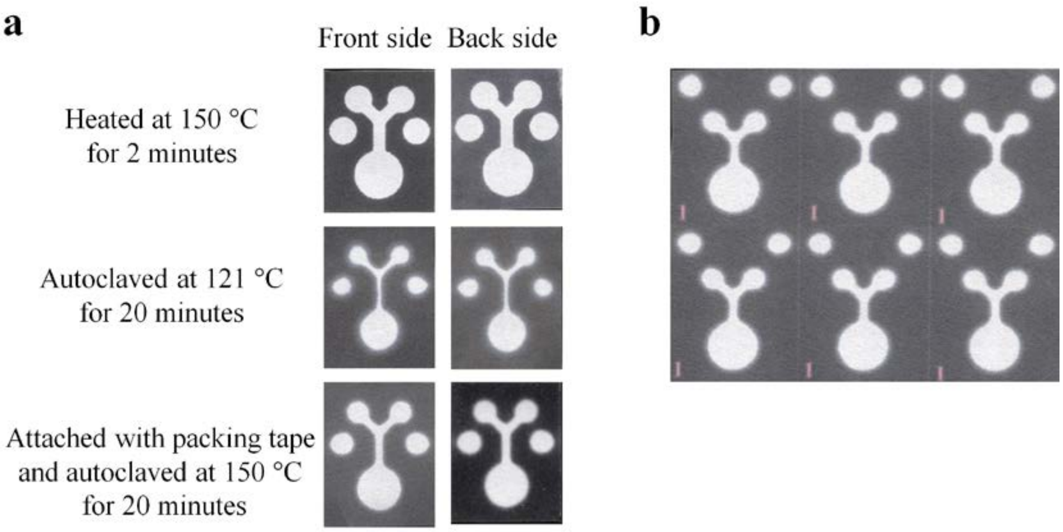

Figure S2. The hydrophilic area on the paper-based analytical devices. A) Representative images of the hydrophilic area after being heated or autoclaved without or with packing tape underneath. B) Reproducibility of the hydrophilic area after sterilization by autoclaving

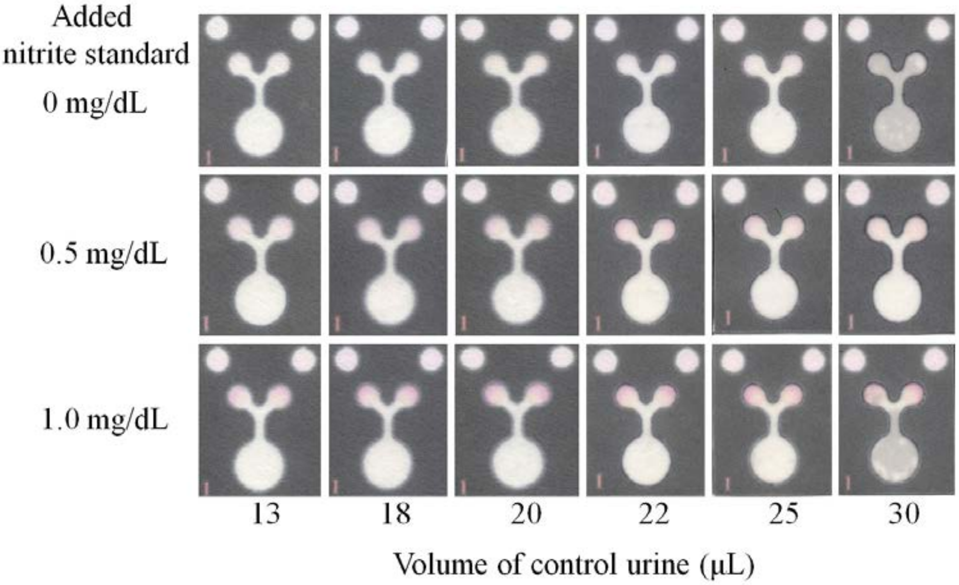

Figure S3. Images show the spread of various volumes of a urine control spiked with a nitrite standard at concentrations of 0, 0.5, 1.0 mg/dL. The PADs were tested with the urine control at volume 13, 18, 20, 22, 25 and 30 µL for 10 minutes of incubation time.

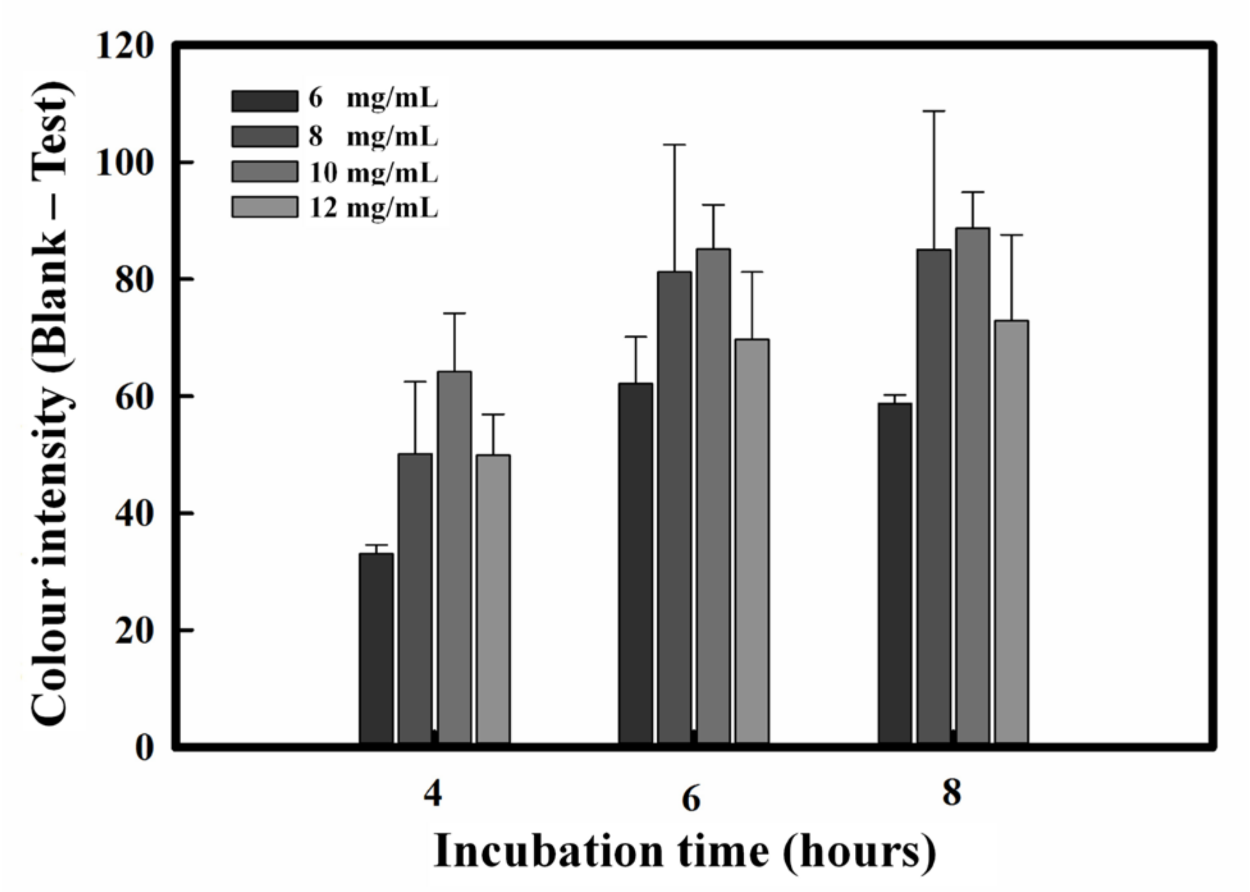

Figure S4. Effect of the X-GlcA substrate concentration and incubation time on the cultivation and identification of *E.coli* using the proposed device (n = 4).

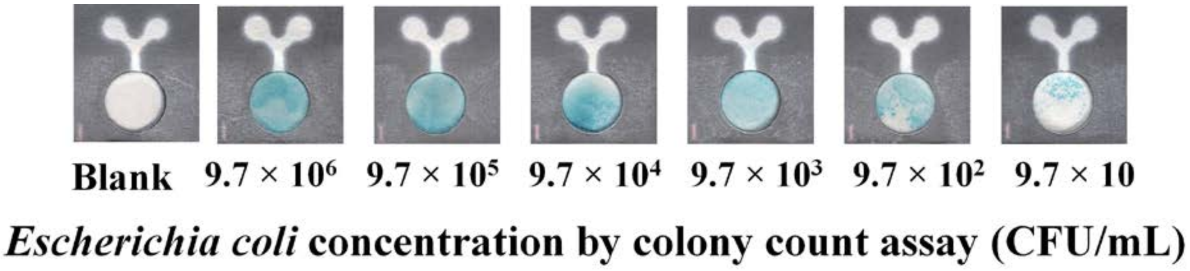

Figure S5. The colour change in the presence of various concentrations of *E. coli* when incubated on the proposed device for 10 hours.

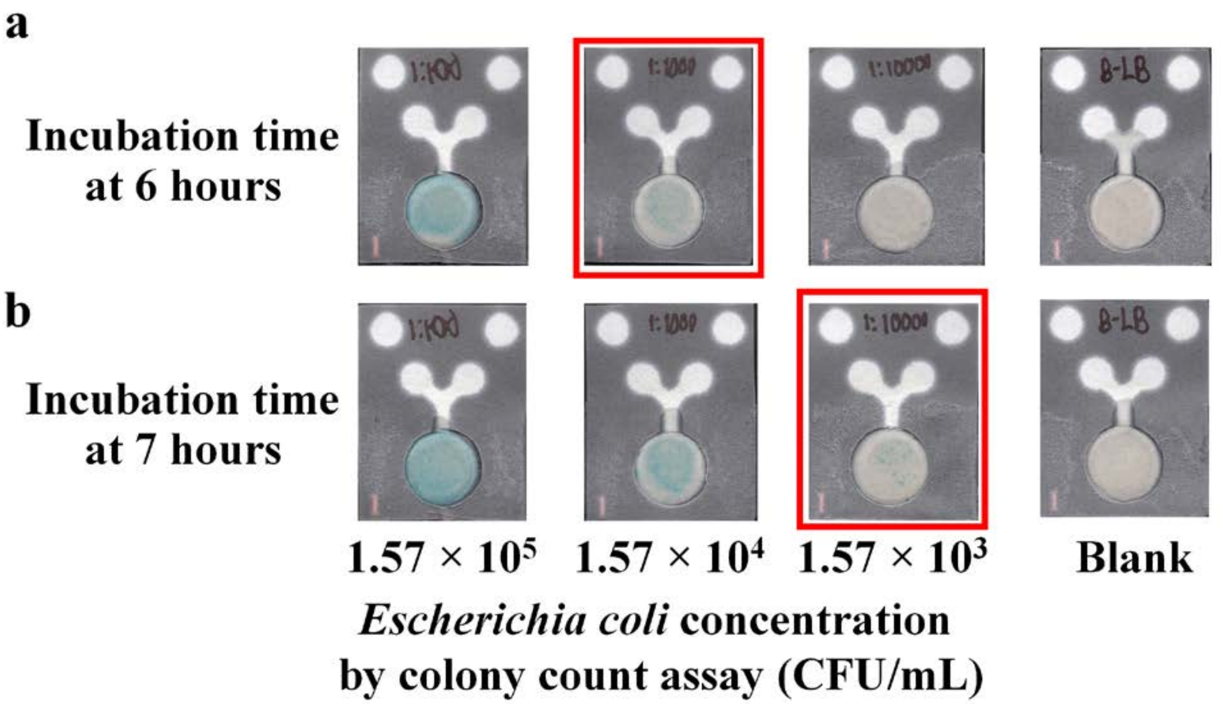

Figure S6. The colour change in the presence of various concentrations of *E. coli* when incubated on the proposed device for 6 hours (a) and 7 hours (b). The red border lines indicate the minimum concentrations of *E.coli* with specified incubation times.
